# A Domain of Herpes Simplex Virus pU_L_33 Required To Release Monomeric Viral Genomes from Cleaved Concatemeric DNA

**DOI:** 10.1128/JVI.00854-17

**Published:** 2017-09-27

**Authors:** Kui Yang, Xiaoqun Dang, Joel D. Baines

**Affiliations:** Department of Pathobiological Sciences, School of Veterinary Medicine, Louisiana State University, Baton Rouge, Louisiana, USA; University of California, Irvine

**Keywords:** herpes simplex virus, U_L_33, DNA cleavage/packaging

## Abstract

Monomeric herpesvirus DNA is cleaved from concatemers and inserted into preformed capsids through the actions of the viral terminase. The terminase of herpes simplex virus (HSV) is composed of three subunits encoded by U_L_15, U_L_28, and U_L_33. The U_L_33-encoded protein (pU_L_33) interacts with pU_L_28, but its precise role in the DNA cleavage and packaging reaction is unclear. To investigate the function of pU_L_33, we generated a panel of recombinant viruses with either deletions or substitutions in the most conserved regions of U_L_33 using a bacterial artificial chromosome system. Deletion of 11 amino acids (residues 50 to 60 or residues 110 to 120) precluded viral replication, whereas the truncation of the last 10 amino acids from the pU_L_33 C terminus did not affect viral replication or the interaction of pU_L_33 with pU_L_28. Mutations that replaced the lysine at codon 110 and the arginine at codon 111 with alanine codons failed to replicate, and the pU_L_33 mutant interacted with pU_L_28 less efficiently. Interestingly, genomic termini of the large (L) and small (S) components were detected readily in cells infected with these mutants, indicating that concatemeric DNA was cleaved efficiently. However, the release of monomeric genomes as assessed by pulsed-field gel electrophoresis was greatly diminished, and DNA-containing capsids were not observed. These results suggest that pU_L_33 is necessary for one of the two viral DNA cleavage events required to release individual genomes from concatemeric viral DNA.

**IMPORTANCE** This paper shows a role for pU_L_33 in one of the two DNA cleavage events required to release monomeric genomes from concatemeric viral DNA. This is the first time that such a phenotype has been observed and is the first identification of a function of this protein relevant to DNA packaging other than its interaction with other terminase components.

## INTRODUCTION

Individual herpes simplex virus (HSV) genomes are composed of covalently linked long (L) and short (S) components that are each flanked by inverted repeats (1, 2). During replication, viral DNA accumulates as concatemers in the nuclei of infected cells. Packaging of individual genomes requires multiple activities of the viral terminase, including the recognition of packaging sequences within terminal *a* sequences of the concatemer, cleavage at the end of the long component, binding the portal vertex in preformed capsids, hydrolysis of ATP to provide energy to pump DNA into the capsid, and scanning of the DNA for the next *cis*-acting sequence in the proper orientation at the S component terminus, followed by its precise cleavage (3–8).

Four types of capsids accumulate in infected cells. The roughly spherical procapsids are the precursors of the other three capsid types and have a porous outer shell and an internal scaffold (9). The B capsid contains an outer shell, which is also present in A and C capsids, and an inner scaffold layer; A capsids lack the internal layer, and the C capsid contains DNA in place of the inner scaffold. A capsids are believed to be the result of aborted packaging events in which the inner shell was expelled or degraded and DNA was not inserted (10, 11).

All herpesviruses encode an enzyme called the terminase; in HSV, the terminase consists of three subunits encoded by the genes U_L_15, U_L_28, and U_L_33 (12–19). The U_L_28 protein (pU_L_28) has been shown to bind Pac1 DNA, which is important for the generation of short component termini (20), whereas U_L_15 has nonspecific cation-dependent nuclease activity *in vitro* and contains an essential Walker box motif, suggesting an ability to hydrolyze ATP (21). The U_L_89-encoded homolog of cytomegalovirus retains the ATPase motif and cation-dependent nuclease activity (22, 23). The structures of the C-terminal portions of the HSV-1 U_L_15 gene product (pU_L_15) and human cytomegalovirus (HCMV) pU_L_89 are virtually superimposable and reveal a conserved RNase H-like structure seen in a number of nucleases and bacteriophage terminase subunits responsible for endonucleolytic DNA cleavage (18, 21, 24).

Cleavage of viral DNA requires both intact capsids and all terminase components (18, 25–27). Mutations outside the ATP binding and nuclease active sites in pU_L_15 can prevent DNA packaging but do not preclude DNA cleavage or the release of intact genomes from concatemeric DNA, indicating that pU_L_15 has a packaging function separate from its nuclease activity (28). These observations are consistent with its roles as both a packaging motor and an endonuclease. pU_L_28, on the basis of its sequence-specific DNA binding activity, may act to restrict pU_L_15's nuclease activity to correct sites at genomic termini.

The predominant model to explain HSV DNA packaging proposed that cleavage at the long terminus is followed by packaging and scanning of DNA until the short terminus is encountered in the concatemer, at which time it is cleaved (29, 30). The *a* sequences that signal cleavage contain subsequences designated DR1-Uc-DR4_*m*_-DR2_*n*_-Ub-DR1, where DR indicates a direct repeat and *m* and *n* indicate the number of repeats (31, 32). Ub and Uc are two unique sequences. Ub has been designated Pac1, and Uc has been designated Pac2. Although two cleavage events that are required to release genomes from the concatemer occur at different times and require different *cis*-acting sequences, they cleave DR1 identically, leaving single 3′ overhangs with 18 bp of DR1 at the L terminus and a single base pair of DR1 at the S terminus (32). The initial cleavage renders the S terminus on the concatemer nonfunctional because it removes much of DR1. Thus, it is believed that only the L terminus is packaged, whereas the S terminus generated from the first cleavage event is degraded. While most genomes in the concatemer are separated by a single *a* sequence, some genomic junctions and L component termini contain multiple *a* sequences that share an intervening DR1 region. During packaging, these tandem *a* sequences presumably pass by the docked terminase and remain uncleaved. Scanning continues until DR1 in the proper orientation is cleaved to generate the S component terminus of DNA to be packaged and to release the genome from the concatemer. The idea that the S component terminus is packaged last is supported by the observations that (i) the S terminus in packaged DNA is most susceptible to DNase digestion and (ii) the short component of packaged DNA never bears more than one *a* sequence, suggesting that it is generated when the terminase cleaves the first encountered packaging sequence in the proper orientation after scanning/packaging of the long and short components (7, 11, 33).

The main goal of the present study was to understand the role of pU_L_33 in the packaging reaction. pU_L_33 interacts with pU_L_28 and enhances the pU_L_28-pU_L_15 interaction (13). Extensive mutagenic analyses previously identified regions of U_L_33 important for DNA packaging and the generation of L component termini (34, 35). In extending these analyses, we have identified several novel mutations in a conserved region of pU_L_33 with a positive charge that do not prevent the cleavage of concatemeric DNA but preclude the release of monomeric genomes. These data suggest that the pU_L_33 component of the terminase is necessary for one of the two DNA cleavage events necessary to release unit-length genomes from concatemeric DNA.

## RESULTS

**FIG 1:**
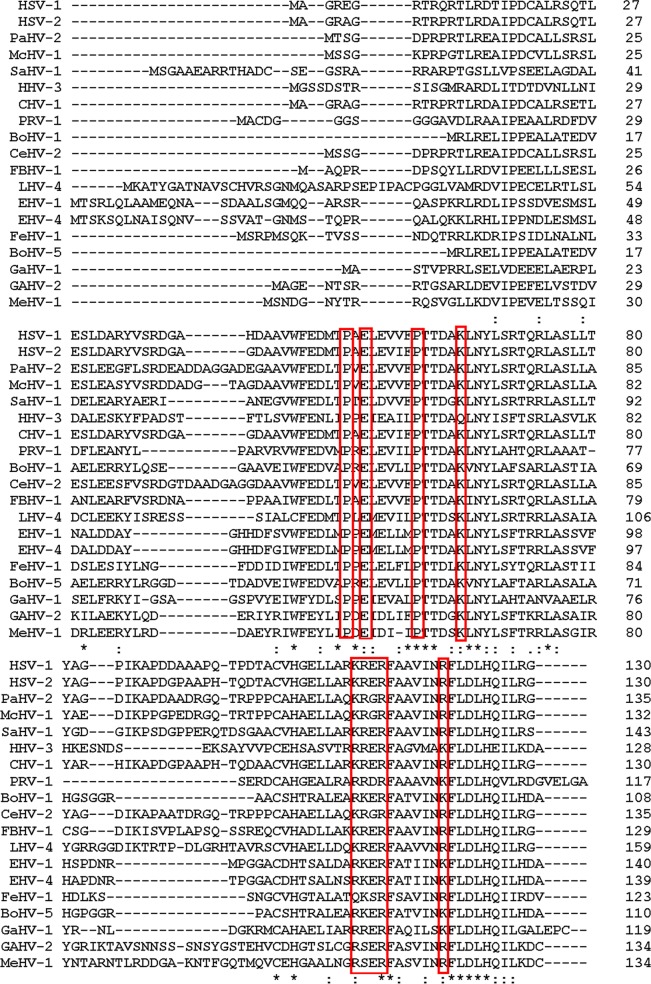
Multiple-sequence alignment of pU_L_33 from 19 alphaherpesviruses. Multiple sequences were aligned with Clustal Omega online software (http://www.ebi.ac.uk/Tools/msa/clustalo/). Conversed residues mutated in this research are highlighted in red boxes. HHV-3, human herpesvirus 3 (varicella-zoster virus); CHV, chimpanzee alphaherpesvirus 1; McHV-1, macacine alphaherpesvirus 1; PaHV-2, papine alphaherpesvirus 2; PRV, pseudorabies virus; BoHV-1, bovine alphaherpesvirus 1; SaHV-1, saimirine alphaherpesvirus 1; CeHV-2, cercopithecine alphaherpesvirus 2; FBHV-1, fruit bat alphaherpesvirus 1; LeHV-4, leporid alphaherpesvirus 4; EHV-1, equid alphaherpesvirus 1; FeHV-1, felid alphaherpesvirus 1; GaHV-1, gallid alphaherpesvirus 1; MeHV-1, meleagrid alphaherpesvirus 1.

In a previous mutagenesis study, the insertion of amino acids VRPQR at position 111 and AAAAA at position 116 of the 130-codon U_L_33 open reading frame precluded the packaging of viral DNA (35). Neither of these insertions precluded interactions with the U_L_28 protein (pU_L_28), although the insertion at codon 116 diminished this interaction, as assessed by a coimmunoprecipitation assay. Alignment of HSV-1 U_L_33 with homologs in other herpesviruses showed that two regions, residues 46 to 77 and 101 to 130, were the most conserved regions of pU_L_33. Basic amino acids were highly conserved at positions corresponding to codons 110 to 113 (encoding KRER in HSV-1). These amino acids were followed by invariant phenylalanine and alanine residues at positions 114 and 115, respectively (Fig. 1).

To investigate whether these conserved regions are important for U_L_33 function, we generated a series of mutant viruses using a bacterial artificial chromosome (BAC) system and assessed their ability to replicate on CV1 cells and complementing cells expressing U_L_33. Consistent with previous work, recombinant viruses with a deletion of residues 50 to 60 or 110 to 120 replicated only in pU_L_33-expressing cells (Table 1). In contrast, a mutant virus lacking codons 120 to 130 replicated in noncomplementing cells at levels similar to those of the wild-type virus (Table 1), suggesting that the last 10 amino acids are dispensable for U_L_33 function.

**TABLE 1:** Phenotype of U_L_33 mutant viruses

Virus	Mutation(s) in pU_L_33	Titer in CV33 cells^*a*^	Titer in CV1 cells	Interaction with pU_L_28^*b*^
33 null	Deletion of pUL33	6 × 10^8^	<10^3^	−
Del50–60	Deletion of residues 50–60	7 × 10^8^	<10^3^	?
Del110–120	Deletion of residues 110–120	1.5 × 10^8^	<10^3^	?
Del120–130	Deletion of residues 120–130	5.5 × 10^8^	2 × 10^8^	+
P52A	Proline to alanine	4 × 10^8^	8 × 10^7^	+
E54A	Glutamic acid to alanine	8 × 10^7^	1 × 10^7^	+
P60A	Proline to alanine	7 × 10^7^	5 × 10^6^	+
K65A	Lysine to alanine	2 × 10^7^	1.2 × 10^7^	+
K86A	Lysine to alanine	2.6 × 10^8^	8 × 10^7^	+
K110A	Lysine to alanine	4 × 10^8^	1.2 × 10^8^	+
R113A	Arginine to alanine	2.2 × 10^8^	4 × 10^7^	+
R120A	Arginine to alanine	9.5 × 10^7^	5 × 10^7^	+
KR	Lysine and arginine at positions 110 and 111 to alanine	5 × 10^8^	<10^3^	+/−
KRER	Lysine and arginine at positions 110, 111, and 113 to alanine	2 × 10^8^	<10^3^	+/−

a Stock viruses were propagated in CV33 cells, while virus titers were determined separately in CV33 or CV1 cells. Virus titers are expressed as PFU per ml.

b The interaction between pU_L_28 and pU_L_33 was determined by coimmunoprecipitation followed by immunoblotting. −, no interaction; ?, unknown; +, interaction; +/−, diminished interaction.

To further investigate the importance of charged residues for pU_L_33 functions, we generated a series of mutant viruses in which basic residues were replaced with alanine, using the recombinant bacterial artificial chromosome system. As shown in Table 1, single-alanine-residue substitutions at positions 52, 54, 60, 65, 85, 110, 113, and 120 reduced the replication of the corresponding mutant viruses by 2- to 14-fold, suggesting modest effects on pU_L_33 function. In contrast, mutants with multiple amino changes (110-KR-111 changed to 110-AA-111 and 110-KRER-113 changed to 110-AAEA-113, designated KR and KRER, respectively), could be propagated only on cells expressing pU_L_33 inasmuch as the ratio of yields from U_L_33-expressing cells infected with these viruses to those from CV1 cells was over 10,000-fold (Table 1). These results were similar to those obtained from analyses of the U_L_33-null mutant performed at the same time (Table 1). We conclude that there was no detectable replication of the KR or KRER mutation in CV1 cells.

**FIG 2:**
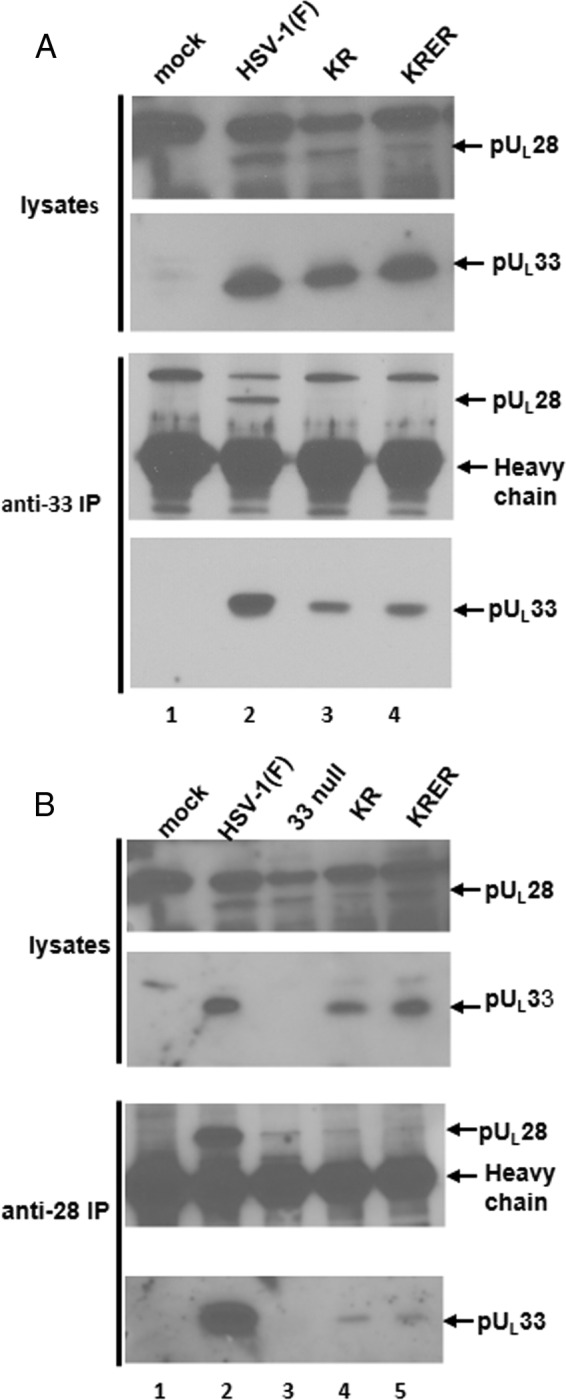
Assessment of pU_L_28 and pU_L_33 interactions in cells infected with wild-type and mutant viruses. CV1 cells were mock infected or infected at an MOI of 5 PFU/cell with HSV-1(F) or U_L_33 mutants, as indicated. The cells were harvested at 18 h postinfection and lysed in RIPA buffer. Precleared lysates were reacted with anti-pU_L_33 (A) or anti-pU_L_28 (B) antibodies. Antigen-antibody complexes were eluted in 2× SDS sample buffer and separated on denaturing 12% polyacrylamide gels. Separated proteins were transferred onto a nitrocellulose membrane and probed with anti-pU_L_28 or anti-pU_L_33 antibodies. Arrows indicate the positions of the indicated proteins and the heavy chain of rabbit immunoglobulin. IP, immunoprecipitation.

To determine if these lethal substitution mutations interfered with the pU_L_28 interaction, cells were mock infected or infected with 5.0 PFU per cell of wild-type HSV-1(F) or U_L_33 mutant viruses. Cells were lysed at 18 h postinfection (p.i.), and clarified lysates were reacted separately with antibody against pU_L_28 or pU_L_33. Antigen-antibody complexes were purified, eluted in SDS-containing buffer, electrophoretically separated on a denaturing SDS-polyacrylamide gel, and transferred onto a nitrocellulose membrane, which was then probed with the same pU_L_28- or pU_L_33-specific antibodies. As shown in Fig. 2, the U_L_33 mutant proteins bearing the 110-AA-111 (KR) and 110-AAEA-113 (KRER) mutations were efficiently immunoprecipitated by the U_L_33 antibody. Unlike wild-type pU_L_33, the mutant proteins failed to coimmunoprecipitate pU_L_28. In the reciprocal interaction, pU_L_28-specific antibody efficiently immunoprecipitated pU_L_33 from cells infected with HSV-1(F), as shown previously (13, 15, 35). Less pU_L_28 was immunoprecipitated with the pU_L_28 antibody from lysates of cells infected with the U_L_33 mutants, consistent with previous results indicating that an optimal interaction of pU_L_33 confers stability to the pU_L_28 protein (13). Despite the lower levels of pU_L_28 in the lysates of cells infected with the U_L_33 mutant viruses, low levels of both mutant U_L_33 proteins were coimmunoprecipitated with the pU_L_28 antibody. We conclude that the U_L_33 mutations diminished the pU_L_28-pU_L_33 interaction but did not completely eliminate it.

**FIG 3:**
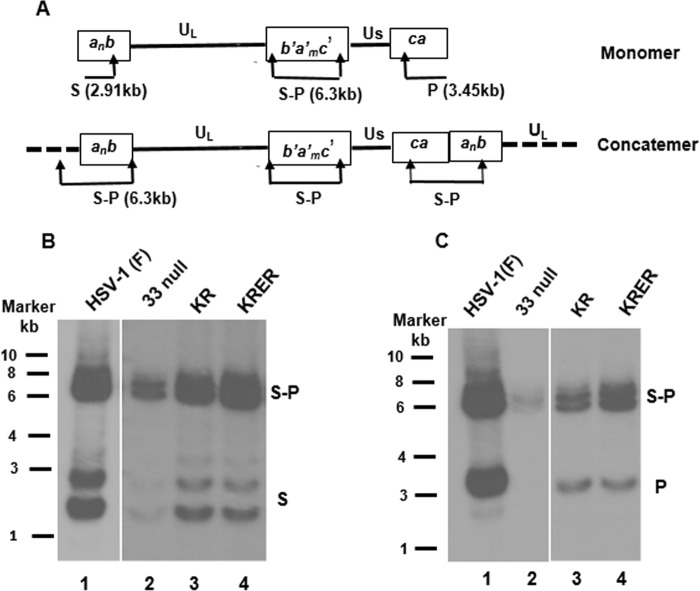
Detection of the ends of the long and short components of HSV DNA by Southern blotting. (A) Schematic diagram of the HSV-1 monomeric genome and concatemeric DNA showing the positions and sizes (in kilobase pairs) of BamHI fragments S, P, and S-P. Arrows indicate the positions of BamHI sites. (B and C) CV1 cells were infected with the indicated viruses, and viral DNAs were extracted 18 h after infection. Viral DNAs were separated on a 0.8% agarose gel, denatured, and transferred onto positively charged nylon membranes. Bound DNAs were probed with radiolabeled DNA representing the terminus of the long component (S fragment of BamHI digestion) (B) or the short component (P fragment) (C) of the viral genome. Fluorographic images were captured by exposing the membrane to X-ray film with intensifying screens at −80°C.

Experiments were then conducted to assess effects of the mutations on the generation of genomic termini. As shown in Fig. 3A, terminase cleavage combined with BamHI cleavage should generate BamHI S fragments of around 2.9 kbp at the L component terminus and a P fragment of 3.45 kbp that reflects the terminus of the S component. Multiple BamHI S fragments were expected because one or more *a* sequences are present at the L termini of different genomes (Fig. 3A). Both the S and P fragments are covalently linked in the S-P fragment derived from the junction of the long and short components in monomeric DNA and the junctions of tandem genomes within concatemeric DNA. Therefore, to test for terminase cleavage activity, viral DNA was purified from cells infected with wild-type and mutant viruses, digested with BamHI, transferred to a positively charged nylon membrane, and probed with radiolabeled fragments specific for the termini of either the long or short components. The results are shown in Fig. 3B.

BamHI S fragments were readily detectable in cells infected with the wild-type virus (Fig. 3B, lane 1) but were hardly detectable in DNA from cells infected with the U_L_33-null virus, indicating that pU_L_33 was necessary for optimal terminase cleavage at the L component terminus. In contrast to these results, S fragments from cells infected with the KR or KREK mutant were readily detectable, indicating that the mutations did not block the cleavage that generates the L component terminus. Similarly, BamHI P fragments representing the S component terminus were readily detected in cells infected with both wild-type and U_L_33 point mutant viruses, but P fragments were barely detected in U_L_33-null-virus-infected cells.

**FIG 4:**
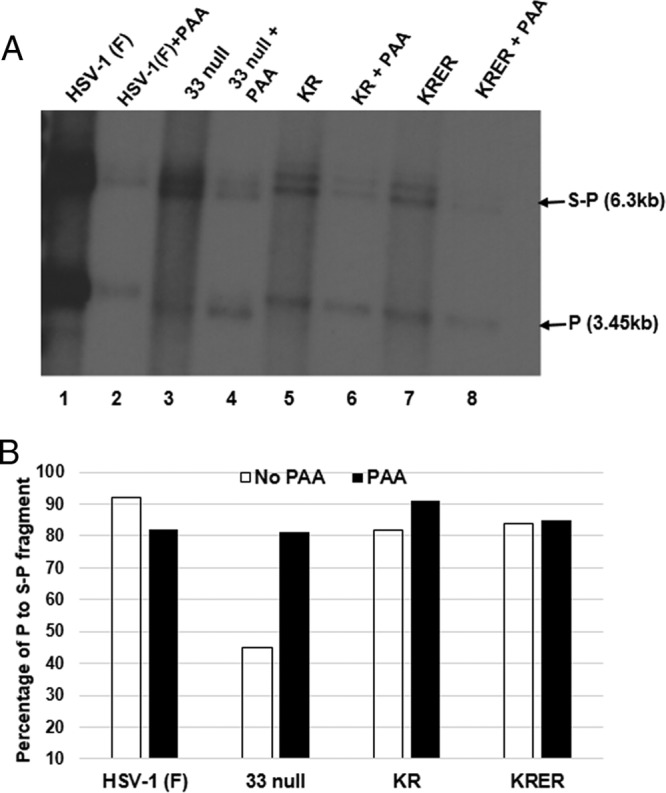
Ratio of terminal to junction BamHI fragments in replicated and input HSV DNA from infected cells. (A) CV1 cells were infected with the indicated viruses at an MOI of 5 PFU per cell in the presence or absence of phosphonoacetic acid (PAA). At 18 h postinfection, viral DNAs were extracted, digested with BamHI, separated on an agarose gel by electrophoresis, denatured, and transferred onto a nylon membrane. Bound DNAs were probed with the denatured radiolabeled P fragment. (B) The levels of the P or S-P fragments in all samples were quantified by using ImageJ software, and the ratios of P fragments to S-P fragments are plotted on a graph.

Because genomic DNA within input virions is linear and would be incorporated into preparations of infected cell DNA, we wanted to ensure that the BamHI S and P fragments that we observed were not due to contaminating input virion DNA. Thus, cells were infected with wild-type and mutant viruses in the presence and absence of phosphonoacetic acid (PAA), a potent viral DNA synthesis inhibitor, and viral DNAs were purified, digested with BamHI, and analyzed on Southern blots probed with the radiolabeled HSV-1(F) P fragment, as detailed above. As shown in Fig. 4, P fragments were detected in input viral DNA in all samples from cells treated with PAA. Moreover, the P fragment was present with the S-P junction fragment at a consistent ratio in all PAA-treated samples. This was expected and reflected the presence of exclusively linear DNA in input virions. Also as expected, the amount of viral DNA in samples from cells infected in the presence of PAA was reduced. Most importantly, the ratios of P to S-P fragments were similar in all samples except those from cells infected with the U_L_33-null mutant in the absence of PAA. In this sample, the P/S-P ratio was reduced by approximately 55%. These data suggest that while pU_L_33 is necessary for the optimal generation of genomic termini, both genomic termini were generated in cells infected with two U_L_33 substitution mutants.

**FIG 5:**
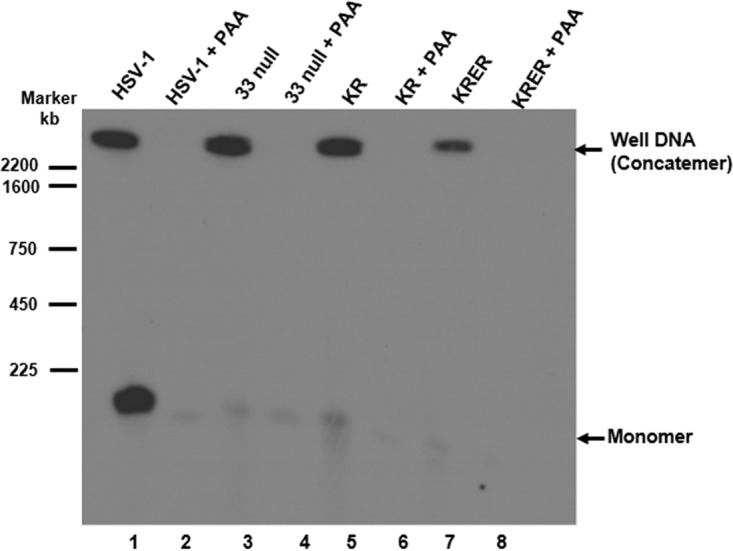
Pulsed-field analysis of viral DNA. CV1 cells were infected with viruses in the presence or absence of PAA, as indicated. Cells were harvested 18 h after infection, washed with PBS, and pelleted by centrifugation. Cell pellets were resuspended in 0.8% agarose and cast into agarose blocks on the bottom of a 10-mm-diameter dish. Viral DNAs in the agarose blocks were separated by pulsed-field electrophoresis. DNA was denatured, transferred onto nitrocellulose, and analyzed by Southern blotting as detailed in Materials and Methods.

To determine whether the terminase cleavage events in U_L_33 mutant viral DNA were sufficient to release genomes from concatemeric viral DNA, we analyzed viral DNA using pulsed-field gel electrophoresis. Cells were infected with HSV-1(F) and U_L_33 mutant viruses in the presence and absence of PAA, and agarose plugs containing the infected cell DNA was subjected to electrophoresis on an agarose gel in a pulsed-field apparatus. The separated DNA was then transferred to a nitrocellulose membrane and probed with radiolabeled viral DNA. As shown in Fig. 5, both concatemeric DNA (well DNA) and monomeric DNAs were detected in untreated HSV-1(F)-infected cells, whereas no concatemeric DNA and small amounts of monomeric DNA were detected after infection in the presence of PAA. We attribute the signal in the PAA-treated sample to input virion DNA. Unlike the results with the wild-type virus, the U_L_33 mutant viruses produced substantially less monomeric DNA than did the wild-type virus inasmuch as virtually all replicated DNA remained in the well, reflecting its presence within DNA concatemers. Small amounts of monomeric DNA were detected in cells infected with U_L_33 mutant viruses in the presence of PAA. Although most of this monomeric DNA signal was attributable to input virion DNA, we could not rule out some generation of monomeric DNA by the U_L_33 mutant viruses because a slightly higher monomeric signal was obtained from cells infected in the absence of PAA than in PAA-treated cells. In the absence of PAA, the monomeric mutant DNA bands were less distinct and exhibited broad bands extending below the migration position of monomeric DNA. This observation suggested that monomeric DNA was cleaved nonspecifically or was partially degraded in these samples.

**FIG 6:**
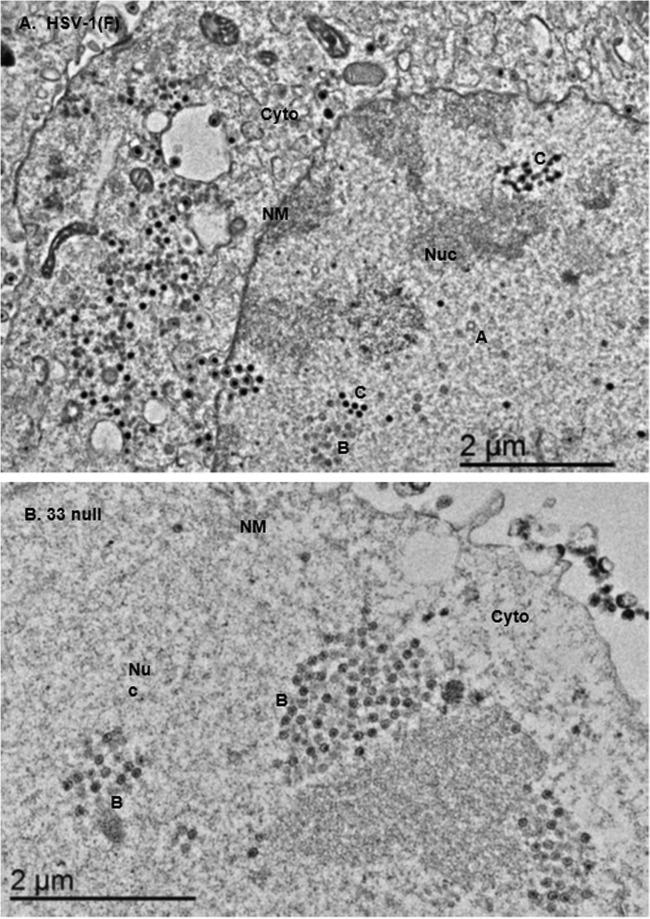
Transmission electron microscopic examination of CV1 cells infected with HSV-1 or U_L_33 mutants. CV1 cells were infected with HSV-1(F) or U_L_33 mutants at an MOI of 5 PFU per cell, fixed at 18 h postinfection, embedded, sectioned, stained with uranyl acetate, and examined with a transmission electronic microscope. A, type A capsid; B, type B capsid; C, type C capsid; NM, nuclear membrane; Cyto, cytoplasm; Nuc, nucleus.

To assess DNA packaging of the mutant viruses, two sets of experiments were conducted. In the first set of these experiments, thin sections of cells infected with wild-type and mutant viruses were examined by electron microscopy. As shown in Fig. 6, HSV-1(F) produced electron-dense capsids containing DNA (type C), capsids with an inner electron-lucent core (type B), and empty capsids (type A). In contrast, while type B capsids were readily detected in cells infected with the U_L_33 mutants, neither type A nor type C capsids were detected in these cells. These results suggested that the U_L_33 mutants exhibited profound defects in DNA packaging.

**FIG 7:**
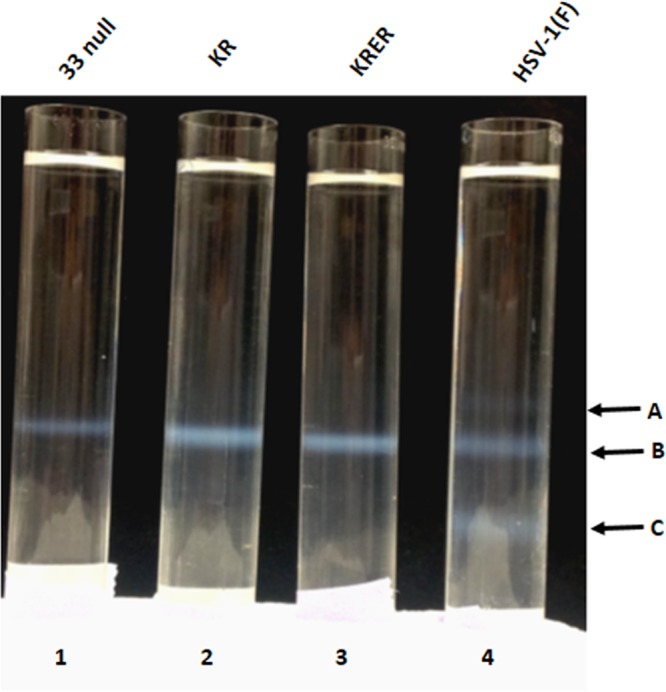
Image of light-refracting capsid-specific bands in continuous 20 to 50% sucrose gradients. CV1 cells were infected with the indicated viruses and lysed 18 h after infection, and capsids were purified by ultracentrifugation on continuous 20 to 50% sucrose gradients. Arrows show the positions of visible light-reflecting bands containing type A, B, and C capsids.

In the second set of experiments, capsids were purified from cells infected with wild-type or mutant viruses and were separated by rate-zonal centrifugation on continuous sucrose gradients. As shown in Fig. 7, only the wild-type virus produced light-refracting bands at positions consistent with all three capsid types, whereas the U_L_33 mutants produced only 1 band consistent with type B capsids that lack DNA. These data further indicated that the U_L_33 mutants were defective in DNA packaging.

## DISCUSSION

To define the function of pU_L_33, we extended mutagenesis studies reported previously, and we report here the generation of new U_L_33 mutants using a bacterial artificial chromosome system. The deletion of the conserved regions between either amino acids 50 and 60 or amino acids 110 and 120 of pU_L_33 impaired its function, and these mutants were able to replicate only in U_L_33-expressing cell lines. Our results are consistent with data from previous work in which pU_L_33 mutations with insertions in these regions were unable to complement the growth of a U_L_33-null virus in transient-packaging assays (35). The last 10 amino acids of pU_L_33 are highly conserved in all alphaherpesviruses but were found to be dispensable for HSV replication.

One goal of this work was to define the region of the pU_L_33 interaction with pU_L_28. Our previous work showed that pU_L_33 bearing a point mutation at position 61 failed to interact with pU_L_28 at a nonpermissive temperature (34). Consistent with this work, the deletion of codons 50 to 60 precluded the interaction of pU_L_33 with pU_L_28 (data not shown), and this mutant virus was unable to replicate in noncomplementing cells (Table 1), suggesting that this region may mediate protein-protein interaction. Surprisingly, mutants with alanine replacements in the conserved residues (52P, 54E, 60P, or 65K) in this region replicated in noncomplementing cells nearly as well as the wild-type virus (Table 1), suggesting that these point mutations did not dramatically affect pU_L_33 function or interactions with pU_L_28. However, we have not tested whether these mutations affect pU_L_33 function at higher temperatures (e.g., 39°C). Whether this conserved region is sufficient to mediate the interaction of pU_L_33 with pU_L_28 needs further study. A virus lacking U_L_33 codons 110 to 120 was also unable to replicate in noncomplementing cells (Table 1). Attempts to assess pU_L_28 interactions with a pU_L_33 mutant lacking codons 110 to 120 were unsuccessful because the deletion of this region precluded detection by the pU_L_33-specific antibody (data not shown). Therefore, it remains unclear whether the deletion of codons 110 to 120 in pU_L_33 affects the interaction with pU_L_28.

Previously characterized terminase component mutants either do not cleave DNA or allow both cleavage events but preclude DNA packaging (11, 18, 26, 28, 36–40). The U_L_33 mutants KR and KRER (Fig. 3 and 4) are unusual because both long and short termini were generated, yet little to no monomeric DNA was released from the concatemer. Because type A capsids that result from aborted packaging events were not detected, it is likely that the U_L_33 mutants abort the packaging reaction after DNA cleavage but before the initiation of DNA packaging. Although there are other possibilities, the simplest explanation for the lack of released monomers is a failure of the second DNA cleavage event. Thus, in noncomplementing cells, the short and long termini of these mutants are generated from a single successful DNA cleavage event, but this is not followed by a second coordinated cleavage event that releases genomes from the concatemer. If true, this would suggest that the first DNA cleavage event always occurs at DR1 in the *a* sequence most distal to U_L_, leaving an S terminus on the concatemer with an *a* sequence bearing a single base pair of DR1.

Indirect evidence also favors the possibility that the KR and KRER mutants are defective in the second DNA cleavage event. Specifically, in coimmunoprecipitation experiments, these UL33 mutants were coimmunoprecipitated with pU_L_28 by anti-pU_L_28 antibodies, but pU_L_28 was not pulled down with the pU_L_33 mutant by anti-pU_L_33 antibodies. These observations suggest that these mutations inhibit the pU_L_33-pU_L_28 interaction but do not completely eliminate it. This imperfect interaction may inhibit proper scanning, which may be required for the coordination of the two cleavage events to release monomeric genomes. The lack of an optimal pU_L_33-pU_L_28 interaction may also preclude the second DNA cleavage event, which is likely augmented by pU_L_28's recognition of Pac1 DNA (20, 41).

It is notable that low levels of L termini are detectable in concatemeric DNA (well DNA) from cells infected with the wild-type virus (42, 43). Concatemeric DNA has been designated well DNA experimentally because it is large enough to be retained in loading wells even after lengthy pulsed-field gel electrophoresis (44). Remarkably, S termini have not been detected in well DNA (44, 45), suggesting that they are absent from concatemeric ends. This observation is surprising because each DNA cleavage event should generate both an S terminus and an L terminus. It is therefore likely that S termini are degraded (when produced from the first terminase DNA cleavage event) or, when produced from the second DNA cleavage event, removed from the concatemer through DNA packaging. Unlike the wild-type virus, the U_L_33 KR and KRER mutants described here produce concatemeric DNA almost exclusively, yet both S and L termini were abundant and readily detected. Taken together, these results suggest that pU_L_33 may also play a direct or indirect role in the degradation of S termini on the concatemer. Another possibility is that both terminase DNA cleavage events occur in the KR and KRER UL33 mutants, but the monomers are somehow retained with the concatemer in well DNA. We do not favor this possibility because monomers are released when well DNA is digested with SpeI, a restriction enzyme that cuts each genome within the concatemer only once (44). Thus, tandem cleavages a genome apart should be sufficient to release monomeric genomes. It follows that the cleavages that we detected in the KR and KRER mutants are much farther apart than genome length, suggesting a discoordination of the two cleavage events relevant to DNA packaging.

In summary, these data reveal previously unknown functions of pU_L_33 and support its role as an important terminase component.

## MATERIALS AND METHODS

### Cells, viruses, and plasmids.

CV1 cells and CV1-derived complementing cells expressing U_L_33 (designated CV33) were described previously (13). The wild type F strain of HSV-1 [HSV-1(F)] and the U_L_33 deletion virus were described previously (46, 47). The HSV-1(F) bacterial artificial chromosome (HSV-BAC) was obtained from Y. Kawaguchi, University of Tokyo (48), and all the recombinant mutants were derived from this HSV-BAC. The pEPKan-S plasmid containing *aphA1* (encoding kanamycin resistance) was a gift from Klaus Osterrieder, University of Berlin. Plasmid pCAGGS-nlsCre expressing Cre recombinase was a gift from Michael Kotlikoff, Cornell University. Bacterial Escherichia coli strain GS1783, used to maintain the HSV-BAC, was obtained from Greg Smith, Northwestern University.

### Generation of recombinant viruses.

All the recombinant viruses were generated with the BAC system as described previously by Tischer at al (49). The primers used to construct recombinant BACs are listed in Table 2. Detailed procedures to generate recombinant BACs were described above, and the expected mutations were confirmed by DNA sequencing. The corresponding recombinant viruses were reconstituted by cotransfecting BAC DNA with the Cre expression plasmid into CV33 cells. Stock viruses of these mutants were prepared in CV33 cells, and the virus titers in CV33 and CV1 cells were determined by using a plaque assay.

**TABLE 2:**
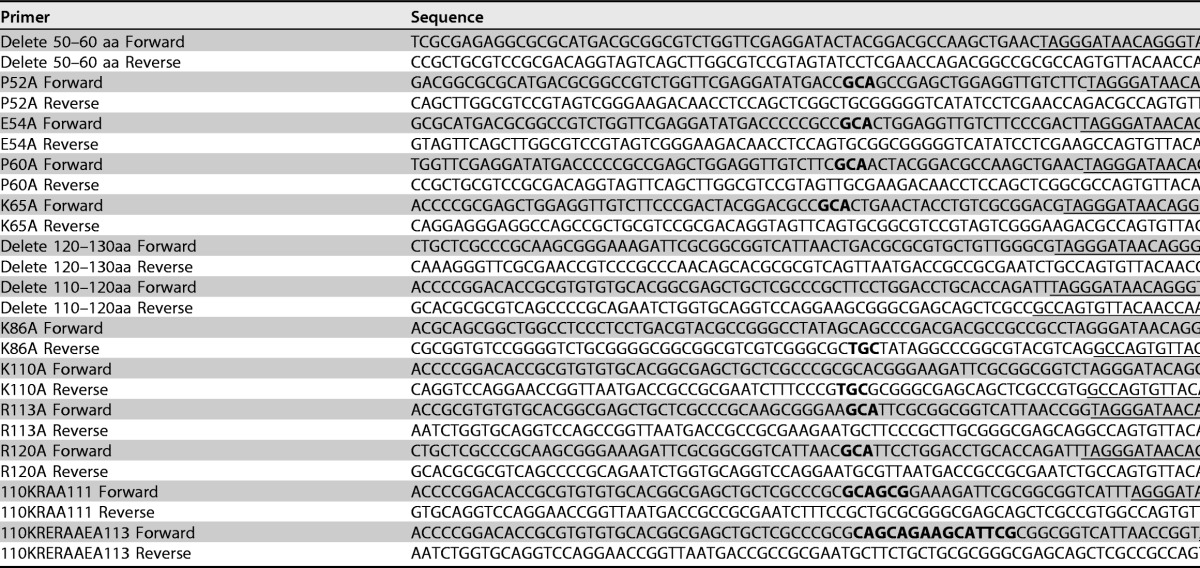
Primers used for generating recombinant viruses^*a*^

a Letters in boldface type represent introduced mutations, and underlined sequences prime to the 5′ or 3′ termini of the kanamycin gene of pEPKan-S.

### Coimmunoprecipitation and Western blotting.

CV1 cells were infected with the indicated viruses at a multiplicity of infection (MOI) of 5.0 PFU per cell. Eighteen hours after infection, cells were washed with phosphate-buffered saline (PBS) and lysed in cold radioimmunoprecipitation assay (RIPA) buffer (50 mM Tris-HCl [pH 7.4], 150 mM NaCl, 1% NP-40, 0.25% sodium deoxycholate, 1 mM EDTA, and a protease inhibitor cocktail). The lysates were clarified by centrifugation at 14,000 rpm for 10 min at 4°C, and the precleared lysates were reacted with anti-pU_L_28 or anti-pU_L_33 antibodies, followed by the addition of Gamma Bind Sepharose beads for immunoprecipitation. Immune complexes bound on the beads were washed extensively with RIPA buffer, eluted in 2× Laemmli sample buffer (Bio-Rad), separated on an SDS–12% polyacrylamide gel, and transferred onto a nitrocellulose membrane for immunoblotting. Both anti-pU_L_28 and anti-pU_L_33 antibodies were diluted at 1:100 for immunoprecipitation and at 1:1,000 for immunoblotting. Horseradish peroxidase-conjugated anti-rabbit immunoglobulin G and enhanced chemiluminescence were used for immunodetection.

### Southern blotting.

CV1 cells were infected with wild-type HSV-1 or U_L_33-null mutant viruses at an MOI of 5 PFU per cell in the presence or absence of 300 μg/ml of PAA. At 18 h postinfection, viral DNA was extracted as described previously (28). Briefly, cells were lysed in 1% NP-40 and digested with proteinase K, followed by phenol-chloroform extraction and ethanol precipitation. Viral DNAs were digested with BamHI and electrophoretically separated on a 0.8% agarose gel. The separated DNAs were denatured with a denaturation solution (1.5 M NaCl, 0.5 M NaOH), neutralized with neutralization buffer (1 M Tris-HCl [pH 8.0], 1 mM NaCl), and transferred onto a positively charged nylon membrane. Bound DNAs were cross-linked with a UV cross-linker (FB-UVXL-1000; Fisher Scientific) and hybridized with denatured ^32^P-labeled BamHI P or S fragments of HSV-1 DNA. The membrane was washed extensively with 1× SSC (1× SSC is 0.15 M NaCl plus 0.015 M sodium citrate), and the positions of the bound probe were determined by fluorography using X-ray film exposed at −80°C in the presence of an intensifying screen. The film was scanned and processed with Adobe Photoshop software, and the signal intensity was quantified with ImageJ software.

### Pulsed-field gel electrophoresis.

Pulsed-field gel electrophoresis experiments were carried out as described previously (28). Briefly, about 3.2 × 10^6^ CV1 cells in a 60-mm-diameter dish were infected with the indicated viruses at an MOI of 5 PFU per cell in the presence or absence of 300 μg/ml PAA for 18 h. Cells were collected in cold PBS and pelleted by spinning them at 4,000 rpm for 5 min in a refrigerated tabletop centrifuge. The pellets were resuspended in 150 μl of PBS and mixed with 300 μl of 1.2% agarose, and the mixture was poured onto the bottom of a 10-mm-diameter dish to form an agarose block. The agarose block was digested with 100 μg/ml proteinase K in digestion buffer (10 mM Tris [pH 8.0], 100 mM EDTA, 1% [wt/vol] *N*-lauroylsarcosine sodium salt [Sarkosyl]) for 20 h at 37°C and washed with storage buffer (10 mM Tris [pH 8.0], 10 mM EDTA). Roughly equally sized agarose plugs were sliced and loaded into the wells of a 0.8% agarose gel, and the wells were sealed with 0.8% low-melting-point agarose. The gel was run in 0.5× TBE buffer (1× TBE buffer contains 89 mM Tris, 89 mM boric acid, and 2 mM EDTA [pH 8.0]) at 6 V/cm for 16 h at 14°C, with an angle of 120° and a pulse time of 45 to 70 s, with a Bio-Rad CHEF-DR II pulsed-field electrophoresis system. After electrophoresis, the gel was soaked in 0.25 M HCl for 45 min to depurinate the DNAs, and the DNAs were further denatured, neutralized, and transferred onto a positively charged nylon membrane as described above. DNAs were UV cross-linked to the membrane and hybridized with the denatured ^32^P-labeled BamHI P fragment of the HSV-1 genome as described above. The bound probe was revealed by exposure of the membrane to X-ray film at −80°C in the presence of intensifying screens.

### Electron microscopy.

Electron microscopic examination of infected cells was performed at the Shared Instrument Facility at Louisiana State University. Confluent CV1 cells in T25 flasks were infected with wild-type HSV-1 or U_L_33 mutants for 18 h. Medium was removed, and the cells were incubated with a fixative solution (2% formaldehyde and 2.5% glutaraldehyde in 0.1 M phosphate buffer [pH 7.0]) for 10 min and collected by scraping into a centrifuge tube, followed by shaking for 2 h. Cells were pelleted, and the supernatant was discarded. The pellets were mixed with equal volumes of 3% agarose and transferred onto a glass slide before solidification. The agarose cubes were rinsed 5 times (15 min each time) with 0.1 M phosphate buffer (pH 7.4) containing 0.08 M glycine, followed by fixing the cells for 1 h in the dark with 2% osmium tetroxide prepared in 0.1 M phosphate buffer (pH 7.4). After 3 washes with distilled water, samples were dehydrated with a graduated series of ethanol concentrations (50%, 70%, 80%, 90%, and 100%, for 15 min under each concentration). This was followed by infiltration with a 1:1 mixture of ethanol (EtOH) and LR White for 2 h and with 100% LR White for 2 h. Samples were dispersed into the bottom of a Beem capsule, and the resin was polymerized at 65°C for 24 h. Ultrathin sections were cut on a Leica EM UC7 microtome, and thin sections (90 nm thick) were collected on 300-mesh nickel grids. Thin sections were counterstained with 2% uranyl acetate for 20 min and then with lead citrate for 7 min. Stained grids were viewed with a JEOL JEM-1400 transmission electron microscope. Images were captured digitally and processed with Adobe Photoshop software.

### Capsid purification.

Capsids were purified from virus-infected cells as described previously (50). About 8 × 10^7^ CV1 cells (four 150-mm dishes) were infected with wild-type HSV-1 or U_L_33 mutant viruses at an MOI of 5.0 PFU per cell. At 18 h postinfection, cells were collected, lysed in lysis buffer (20 mM Tris-HCl [pH 7.6], 500 mM NaCl, 1% Triton X-100, 1 mM EDTA, 1 mM dithiothreitol, and a protease inhibitor), and precleared by centrifugation at 10,000 rpm for 20 min. The precleared lysates were loaded onto a 5-ml 35% (wt/vol) sucrose cushion and pelleted by spinning in a Beckman SW28 rotor at 24,000 rpm for 1 h. Capsids were resuspended in 600 μl TNE buffer (20 mM Tris-HCl [pH 7.6], 500 mM NaCl, 1 mM EDTA) and loaded onto a continuous 20% to 50% sucrose gradient, followed by centrifugation at 24,500 rpm for 1 h. After centrifugation, the light-refracting capsids were photographed.
